# P18 Treatment patterns and extent of inappropriate antimicrobial treatment among female patients with uncomplicated urinary tract infection in England

**DOI:** 10.1093/jacamr/dlad077.022

**Published:** 2023-08-02

**Authors:** Mark Wilcox, Dave Heaton, Aruni Mulgirigama, Ashish V Joshi, Viktor Chirikov, Daniel C Gibbons, David Webb, Xiaocong L Marston, Myriam Alexander, Fanny S Mitrani-Gold

**Affiliations:** University of Leeds & Leeds Teaching Hospitals, Leeds, UK; OPEN Health, Marlow, UK; GSK, Brentford, London, UK; GSK, Collegeville, PA, USA; OPEN Health, Bethesda, MD, USA; GSK, Brentford, London, UK; GSK, Brentford, London, UK; OPEN Health, Marlow, UK; OPEN Health, Marlow, UK; GSK, Collegeville, PA, USA

## Abstract

**Background and Objectives:**

Inappropriate antimicrobial treatment of uncomplicated urinary tract infection (uUTI) has potential impacts on healthcare resource use and costs. We characterized treatment patterns for uUTI in England, including the extent of inappropriate treatment.

**Methods:**

This retrospective cohort study utilized anonymized patient data from the Clinical Practice Research Datalink (CPRD) database from 1 January 2018 to 31 December 2019. Female patients ≥12 years with a diagnosis of community-acquired uUTI (index), who had received ≥1 oral antibiotic within ±5 days of index and had ≥14 months of CPRD data linked to English Hospital Episode Statistics data, were included. Patients who attended an accident and emergency department for urologic reasons, were hospitalized 28 days pre-index, or had complicated UTI or complicating comorbidities were excluded. uUTI episodes were defined as the 28-days post-index and follow-up data was captured through 29 February 2020, for a potential episode extension due to re-prescription. Treatment appropriateness for index uUTI in terms of drug class, duration and dose, was defined per NICE UTI guidelines (Figure 1).

**Figure 1.**
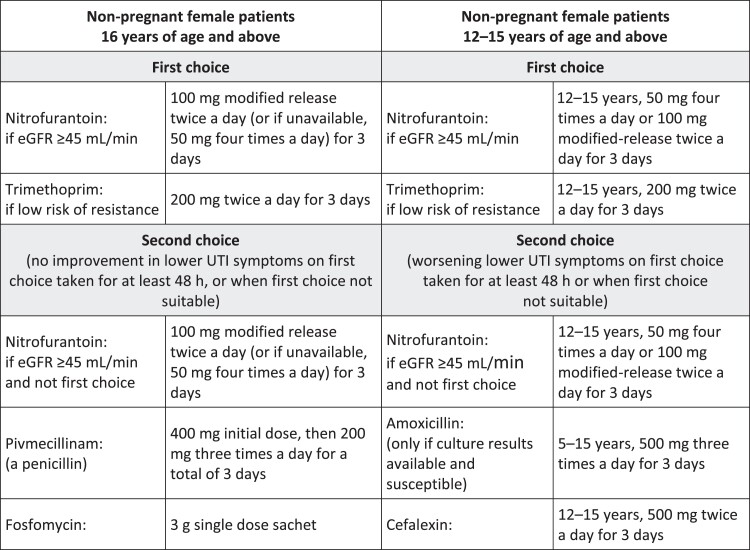
NICE recommendations for antibiotic treatment for non-pregnant female patients per NG109. This guideline was published on 31 October 2018, i.e. midway through the data/case review period. eGFR, estimated glomerular filtration rate; NG, NICE Guideline.

**Results:**

Overall, 120519 female uUTI patients were evaluated. First-line therapy among 118540 female patients ≥16 years of age included nitrofurantoin (71%), trimethoprim (14.5%), amoxicillin (4%), pivmecillinam (2.7%) and fluoroquinolone (1.6%), with evident variation in treatment by age and line of therapy (Table 1). Inappropriate treatment, i.e. per above definition, was given to 52460 (43.5%) patients, and was less common among patients with re-prescription (35%) than without re-prescription (44%). Patients requiring a re-prescription were older, more often menopausal, had greater comorbid burden, and had higher prevalence of previous recurrent uUTI or prior exposure to antimicrobials (Table 2). Few regional or practice-level differences were observed (Table 2).

**Table 1.** Most common antibiotic treatment at each line of therapy (%), among non-pregnant female patients by age group (aged ≥16 years and 12–15 years)

Single-agent therapies/Metric	≥16 years old, *N* (%)		
	1L *N*=118540 (100)	2L *N*=31425 (26.5)	3L *N*=6672 (5.6)
Nitrofurantoin			
*n* (%)	84407 (71.2)	11741 (37.4)	1777 (26.6)
Duration, mean (SD), median [IQR]	4.26 (2.88), 3 [3–5]	5.58 (6.10), 5 [3–7]	6.88 (9.00), 5 [3–7]
Trimethoprim			
*n* (%)	17187 (14.5)	6199 (19.7)	1093 (16.4)
Duration, mean (SD), median [IQR]	4.81 (4.5), 3 [3–7]	6.22 (7.12), 5 [3–7]	8.71 (11.51), 7 [3–7]
Pivmecillinam hydrochloride			
*n* (%)	3201 (2.7)	1924 (6.1)	459 (6.9)
Duration, mean (SD), median [IQR]	21.91 (10.43), 28 [8–28]	21.68 (10.54), 28 [7–28]	21.26 (10.68), 28 [7–28]
Fosfomycin trometamol			
*n* (%)	365 (0.3)	273 (0.9)	94 (1.4)
Duration, mean (SD), median [IQR]	2.82 (6.42), 1 [1–1]	3.42 (7.21), 1 [1–2]	2.82 (6.04), 1 [1–2]

	12–15 years old, *N* (%)		
Single-agent therapies/Metric	1L *N*=1979 (100)	2L *N*=453 (22.9)	3L *N*=67 (3.4)
Nitrofurantoin			
*n* (%)	1157 (58.5)	168 (37.1)	23 (34.3)
Duration, mean (SD), median [IQR]	3.84 (2.45), 3 [3–3]	4.79 (2.81), 3 [3–7]	4.52 (1.90), 3 [3–7]
Trimethoprim			
*n* (%)	630 (31.8)	115 (25.4)	13 (19.4)
Duration, mean (SD), median [IQR]	4.31 (3.31), 3 [3–5]	5.50 (4.72), 4 [3–7]	6.85 (6.64), 7 [3–7]
Amoxicillin			
*n* (%)	69 (3.5)	40 (8.8)	10 (14.9)
Duration, mean (SD), median [IQR]	6.91 (4.71), 7 [5–7]	7.35 (6.10), 6 [5–7]	10.40 (11.01), 5 [5–7]
Cefalexin			
*n* (%)	41 (2.1)	35 (7.7)	9 (13.4)
Duration, mean (SD), median [IQR]	7.44 (4.96), 7 [5–7]	10.26 (10.81), 7 [5–7]	8.00 (4.69), 7 [7–7]

1L, first-line therapy; 2L, second-line therapy; 3L, third-line therapy.

**Table 2.** Patient-level, region-level and practice-level characteristics for patients with inappropriate index antibiotic treatment overall, and by re-prescription status

Variable description	All patients *N*=120519 (100)	Patients with re-prescription *n*=8019 (6.7)	Patients without re-prescription *n*=112500 (93.3)
Inappropriate antibiotic, *n* (%)	52460 (43.5)	2807 (35.0)	49653 (44.1)
Patient-level characteristics			
Age at diagnosis, *n* (%)			
≥12 to <16	638 (1.2)	21 (0.7)	617 (1.2)
≥16 to <30	8300 (16)	220 (7.8)	8080 (16)
≥30 to <40	7447 (14)	249 (8.9)	7198 (15)
≥40 to <50	6105 (12)	216 (7.7)	5889 (12)
≥50 to <65	10372 (20)	537 (19)	9835 (20)
≥65	19598 (37)	1564 (56)	18034 (36)
Menopausal status, yes, *n* (%),	15670 (30)	1095 (39)	14575 (29)
History of recurrent uUTI, yes, *n* (%)	2987 (5.7)	412 (15)	2575 (5.2)
History of prior exposure to antimicrobial treatment, yes, *n* (%)	34034 (65)	2231 (79)	31803 (64.05)
Mild/moderate renal impairment, *n* (%)Yes	1142 (2.2)	106 (3.8)	1036 (2.1)
Liver disease, yes, *n* (%)	47 (0.1)	<5 (<0.2)	NE
Controlled diabetes, yes, *n* (%)	1200 (2.3)	114 (4.1)	1086 (2.2)
Charlson comorbidity index, mean (SD)	0.22 (0.49)	0.33 (0.58)	0.21 (0.48)
Index uUTI consultation as a home visit, yes, *n* (%)	961 (1.8)	89 (3.2)	872 (1.8)
Number of admissions in prior year, mean (SD)	0.35 (0.85)	0.52 (1.20)	0.34 (0.82)
Total number of days spent in hospital in prior year, mean (SD)	3.55 (11.31)	5.90 (15.60)	3.37 (10.90)
Number of A&E attendances in prior year, mean (SD)	0.55 (1.32)	0.66 (1.44)	0.55 (1.31)
Number of medication classes defined by each of BNF classes in the prior 12 months, *n* (%)			
Gastro-intestinal system	22465 (46)	1590 (58)	20875 (45)
Cardiovascular system	21355 (44)	1580 (58)	19775 (43)
Respiratory system	13009 (27)	835 (31)	12174 (26)
Central nervous system	27791 (57)	1864 (68)	25927 (56)
Infections	34906 (71)	2256 (82)	32650 (70)
Endocrine system	14982 (31)	1082 (40)	13900 (30)
Obstetrics, gynaecology and urinary-tract disorders	14776 (30)	925 (34)	13851 (30)
Malignant disease and immunosuppression	756 (1.5)	58 (2.1)	698 (1.5)
Nutrition and blood	14420 (29)	1043 (38)	13377 (29)
Musculoskeletal and joint diseases	13239 (27)	905 (33)	12334 (27)
Eye	6110 (12)	496 (18)	5614 (12)
Ear, nose and oropharynx	7734 (16)	524 (19)	7210 (16)
Skin	14830 (30)	1057 (39)	13773 (30)
Immunological products and vaccines	6403 (13)	473 (17)	5930 (13)
Anaesthesia	1630 (3.3)	98 (3.6)	1532 (3.3)
Preparations used in diagnosis	0 (0)	0 (0)	0 (0)
Other drugs and preparations	239 (0.5)	25 (0.9)	214 (0.5)
Region-level, *n* (%)			
Quintiles of IMD^a^			
Q1	27749 (23)	2117 (26)	25632 (23)
Q2	24277 (20)	1691 (21)	22586 (20)
Q3	22992 (19)	1527 (19)	21465 (19)
Q4	22765 (19)	1401 (18)	21364 (19)
Q5	22658 (19)	1277 (16)	21381 (19)
Missing	78 (0.1)	6 (0.1)	72 (0.1)
Rural-urban area classification			
Rural	15883 (13)	1244 (16)	14639 (13)
Urban	104636 (87)	6775 (85)	97861 (87)
Practice-level, *n* (%)			
Small practice size (<8000 patients)	31762 (26)	2016 (25)	29746 (26)
Medium practice size (8000–11000 patients)	26189 (22)	1733 (22)	24456 (22)
Large practice size (>11000 patients)	62222 (52)	4258 (53)	57964 (52)

Q1, least deprived; Q5, most deprived. A&E, accident and emergency; IMD, index of multiple deprivation; NE, not estimable value; Q, quintile.

**Conclusions:**

Inappropriate treatment, based on drug class, duration and dose, was common among patients with uUTI, and inappropriate treatment was higher among patients without re-prescription versus those with re-prescription. Patient-level differences, rather than regional- or practice-level differences, appear to drive inappropriate antibiotic use. The effect of inappropriate treatment on disease progression risk will be assessed in future work.

